# Learning Curve of Robotic-Assisted Total Mesorectal Excision for Rectal Cancer

**DOI:** 10.3389/fonc.2022.931426

**Published:** 2022-07-11

**Authors:** Bo Tang, Tao Li, Gengmei Gao, Jun Shi, Taiyuan Li

**Affiliations:** Department of General Surgery, The First Affiliated Hospital of Nanchang University, Nanchang, China

**Keywords:** robotic, learning curve, TME, CUSUM, RA-CUSUM

## Abstract

**Background:**

Although some studies have assessed the learning curve of robotic-assisted total mesorectal excision for rectal cancer, most studies included limited sample sizes, no study used postoperative complications as an independent variable to analyze the learning curve of robotic rectal surgery, and no study evaluated the influence of the learning curve on long-term oncologic outcomes.

**Methods:**

Clinical data on consecutive patients who underwent robotic-assisted total mesorectal excision for rectal cancer by a single surgeon between January 2015 and December 2018 at the First Affiliated Hospital of Nanchang University were retrospectively collected. The cumulative sum (CUSUM) and risk-adjusted cumulative sum (RA-CUSUM) were used to visualize the learning curve of operation time and postoperative complications (CD ≥ grade II). Comparisons of clinical outcomes at different learning phases analyzed by RA-CUSUM were performed after propensity score matching.

**Results:**

A total of 389 consecutive patients were included in the analysis. The numbers of patients needed to overcome the learning curves of operation time and postoperative complications of robotic-assisted laparoscopic surgery for rectal cancer were 34 and 36, respectively. The learning process was divided into two phases based on RA-CUSUM: the learning phase (1st-36th cases) and the mastery phase (37th-389th cases). Before matching, the mastery phase had more patients with older age, lower tumor location, and neoadjuvant therapy. After matching, the two phases exhibited similar characteristics. The operation time, intraoperative blood loss, postoperative hospital stay, and postoperative complications in the mastery phase were reduced compared with the learning phase, with a median follow-up of 35 months, and the long-term oncologic outcomes were not significantly different between the two phases.

**Conclusions:**

An experienced laparoscopic surgeon initially implements robotic-assisted total mesorectal excision for rectal cancer, surgical outcomes improved after 36 cases, and the learning curve seemingly did not have an obvious impact on long-term oncologic outcomes.

## Introduction

Laparoscopy has been widely used in the surgical treatment of rectal cancer; compared with laparotomy, laparoscopic rectal surgery has advantages in reducing intraoperative bleeding and accelerating postoperative recovery (1, 2). However, due to the inherent limitations of laparoscopic platforms, narrow pelvic operations, and high requirements for assistants, laparoscopic rectal cancer surgery is difficult to master (3), and the learning curve for laparoscopic rectal cancer surgery is longer and easily results in a high conversion rate and postoperative complications in the learning phase (4, 5).

Since Pigazzi et al. (6) first reported Da Vinci robot-assisted rectal cancer resection in 2006, the application of robots has gradually increased in the treatment of rectal cancer. Robotic platforms can overcome some limitations of conventional laparoscopic surgery, such as an immersive three‐dimensional view of the surgical field, better dexterity capability, stable camera platform, and improved ergonomics for the surgeon (7). The safety and feasibility of robotic-assisted laparoscopic surgery for rectal cancer have been demonstrated by previously published studies (7–11); subsequently, the learning curve to determine how this technique can be taught to novices is also important to study. Although some studies have reported the learning curve of robotic-assisted laparoscopic surgery for rectal cancer (12–17), most reports included limited sample sizes, which may be inadequate to achieve statistical significance and cannot evaluate the growth process of surgical techniques from a holistic perspective. No study used postoperative complications as an independent variable to analyze the learning curve of robotic-assisted laparoscopic surgery for rectal cancer, and no study evaluated the influence of the learning curve on long-term oncologic outcomes.

Therefore, the primary aim of this study was to analyze the learning curve of postoperative complications of robotic-assisted laparoscopic surgery for rectal cancer. The secondary aim was to assess the learning curve of operation time and to evaluate the influence of the learning curve on long-term oncologic outcomes in robotic-assisted laparoscopic surgery for rectal cancer.

## Materials and Methods

### Patients and Study Design

Clinical records of consecutive patients who underwent robotic-assisted total mesorectal excision for rectal cancer by a single surgeon between January 2015 and December 2018 at the First Affiliated Hospital of Nanchang University were retrospectively reviewed. In this study, the inclusion criteria were as follows: 1) diagnosed rectal cancer; 2) tumor located within 15 cm of the anal verge; and 3) robotic rectal cancer surgery performed. The exclusion criteria were as follows: 1) synchronous surgeries for other organs; 2) distant metastasis; 3) incomplete clinical data; 4) emergency surgery; and 5) other malignant tumor history. This study received ethical approval from the First Affiliated Hospital of Nanchang University.

### Variables

Age, sex, American Society of Anesthesiologists (ASA) score, body mass index (BMI), carcinoembryonic antigen (CEA), CA 19.9, tumor location from the anal verge (measured by preoperative rectal MRI), and neoadjuvant therapy were included in the patient characteristics. Operation method, natural orifice specimen extraction surgery (NOSES), stoma, operation time (skin to skin), intraoperative blood loss (estimated by subtracting the amount of intraoperative flushing fluid from the amount of liquid in the suction bottle, and then adding the weight of blood gauze minus the weight of dry gauze (calculated as 1g = 1ml)), conversions (defined as the use of a laparotomy wound for any part of total mesorectal dissection), postoperative hospital stay, postoperative complications (anastomotic leakage was defined as a defect of the intestinal wall at the anastomosis leading to communication between intra and extra luminal compartments that required an intervention and classified according to the recommendation of International Study Group of Rectal Cancer (18); presacral infection defined as perineal incision infection after abdominoperineal resection), and reoperation were included in the perioperative results. The number of resected lymph nodes, numbers of insufficient number of harvested lymph nodes (less than 12), positive distal resection margin (DRM), positive circumferential resection margin (CRM), and TNM stage (according to the 8th American Joint Committee on Cancer staging system) were included in the postoperative pathological results. The overall survival rate (OS) and disease-free survival rate (DFS) were included in the long-term outcomes.

### Surgical Technique

**Figure 1:**
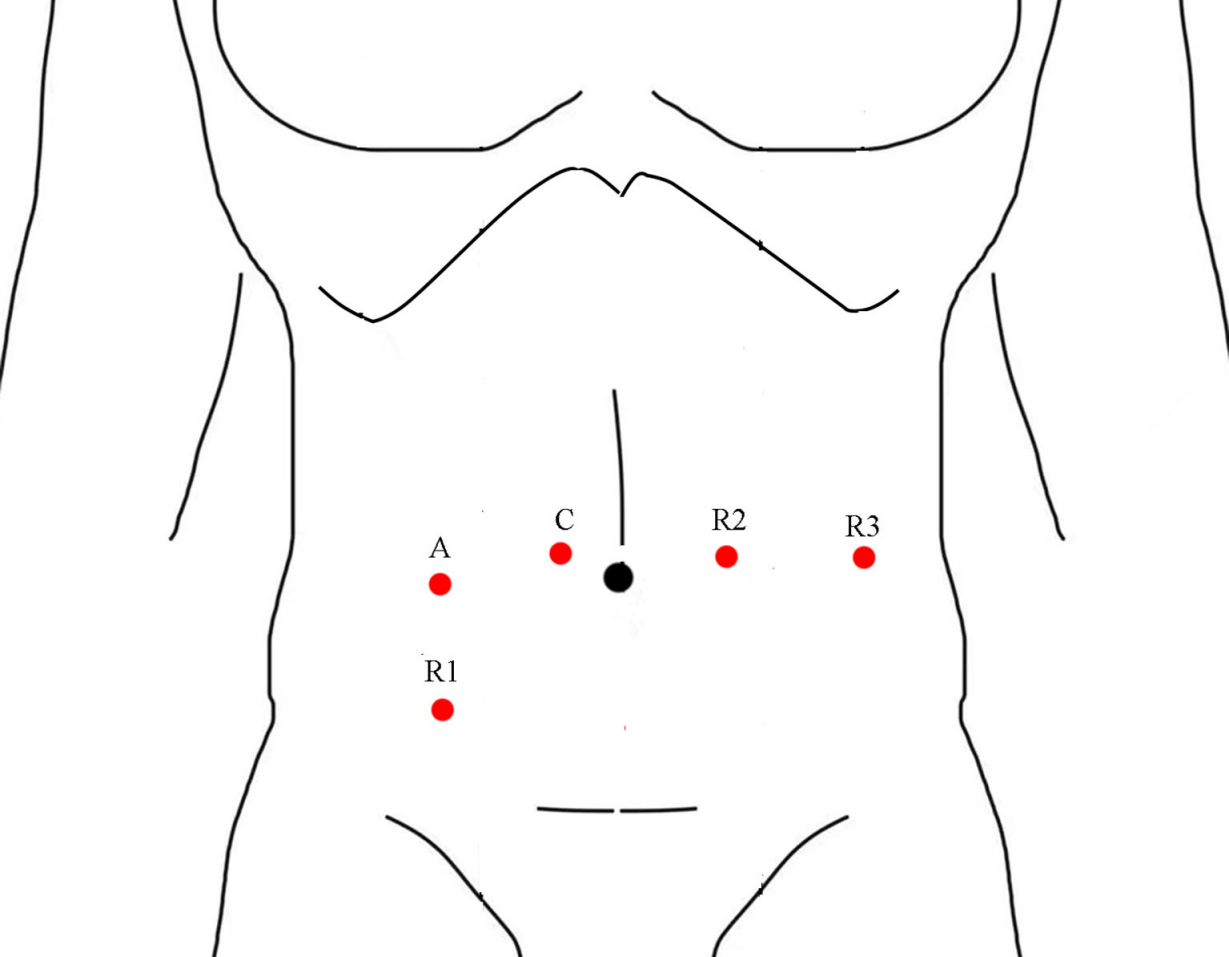
Port placement in robotic rectal cancer surgery.

All operations were completed by one single experienced laparoscopic surgeon, the surgeon had completed thousands of laparoscopic gastrointestinal surgeries and was certified by an international training center (Chinese University of Hong Kong, Prince of Wales Hospital) before implementing robotic-assisted surgery. The da Vinci Surgical System Si was used in all operations. The surgical technique of robotic-assisted total mesorectal excision for rectal cancer and NOSES has been described in detail previously (19, 20). Four robotic ports and one assistant port were placed (**Figure 1**), and all rectal cancer resections were performed with a medial-to-lateral approach and adherence to the total mesorectal resection principle. The specimen was extracted through mini-laparotomy at the lower abdominal or natural orifice (anus and vaginal) in cases of low anterior resection (LAR) or intersphincteric resection. A diverting ileostomy was created based on the quality of the anastomosis, and abdominoperineal resection (APR) was performed if the distal resection margin of 1–2 cm cannot be confirmed with negative in low anterior resection.

### Statistical Analysis

All statistical analyses were performed using SPSS version 22.0 (SPSS Institute, Chicago, IL, USA). The Chi-square test or Fisher’s exact test was used to compare qualitative variables, Student’s t-test or the Mann–Whitney U test was used to analyze quantitative variables between the groups, the Kaplan–Meier method was used to estimate the overall survival rate and disease-free survival rate, and the log-rank test was used to evaluate differences between the groups. P-values <0.05 were considered statistically significant.

### Cumulative Sum

CUSUM was applied to assess the learning curve of operation time. This method accumulated deviation between each individual case and the mean value of the cohort in a sequential manner (21, 22). The equation was defined as $CUSUM=\sum_{i=1}^{n}\left(Xi-u\right)$ , where Xi represents the operation time of each case and u represents the mean operation time of the cohort.

### Risk-Adjusted Cumulative Sum

RA-CUSUM was used to evaluate the learning curve of postoperative complications (CD ≥ II). This method is an extended version of the CUSUM method, which has been confirmed as a useful method resolving bias by balancing each patient’s inherent risk for complications and the surgeon’s maturity during the learning period (23, 24). The equation of this method was defined as $\text{RA}-\text{CUSUM}=\sum_{i=1}^{n}\left(xi-t\right)+{\left(-1\right)}^{xi}Pi$ where Xi=1 represents the occurrence of postoperative complications, Xi=0 indicates no complication, and t represents the observed event rate. Pi represents the predicted probability of postoperative complications calculated by logistic regression analysis. Age, sex, ASA grade, BMI, distance between the tumor and anus, number of resected lymph nodes, tumor size, tumor histology, and the type of specimen extraction were included in the logistic regression analysis.

The locally high and low peaks within the CUSUM and RA-CUSUM curves were used to determine the turning points (TPs), and the TPs were used to differentiate each learning phase.

### Propensity Score Matching

Characteristics including age, sex, American Society of Anesthesiologists score, body mass index, carcinoembryonic antigen, CA 19.9, distance between the tumor and the anal verge, and neoadjuvant therapy were used for propensity score matching. Patients were matched 1:1 between different phases using the nearest neighbor method within the calipers with 0.2 of the standard deviation of the propensity score.

## Results

### Patients and Learning Curve Analysis

**Figure 2:**
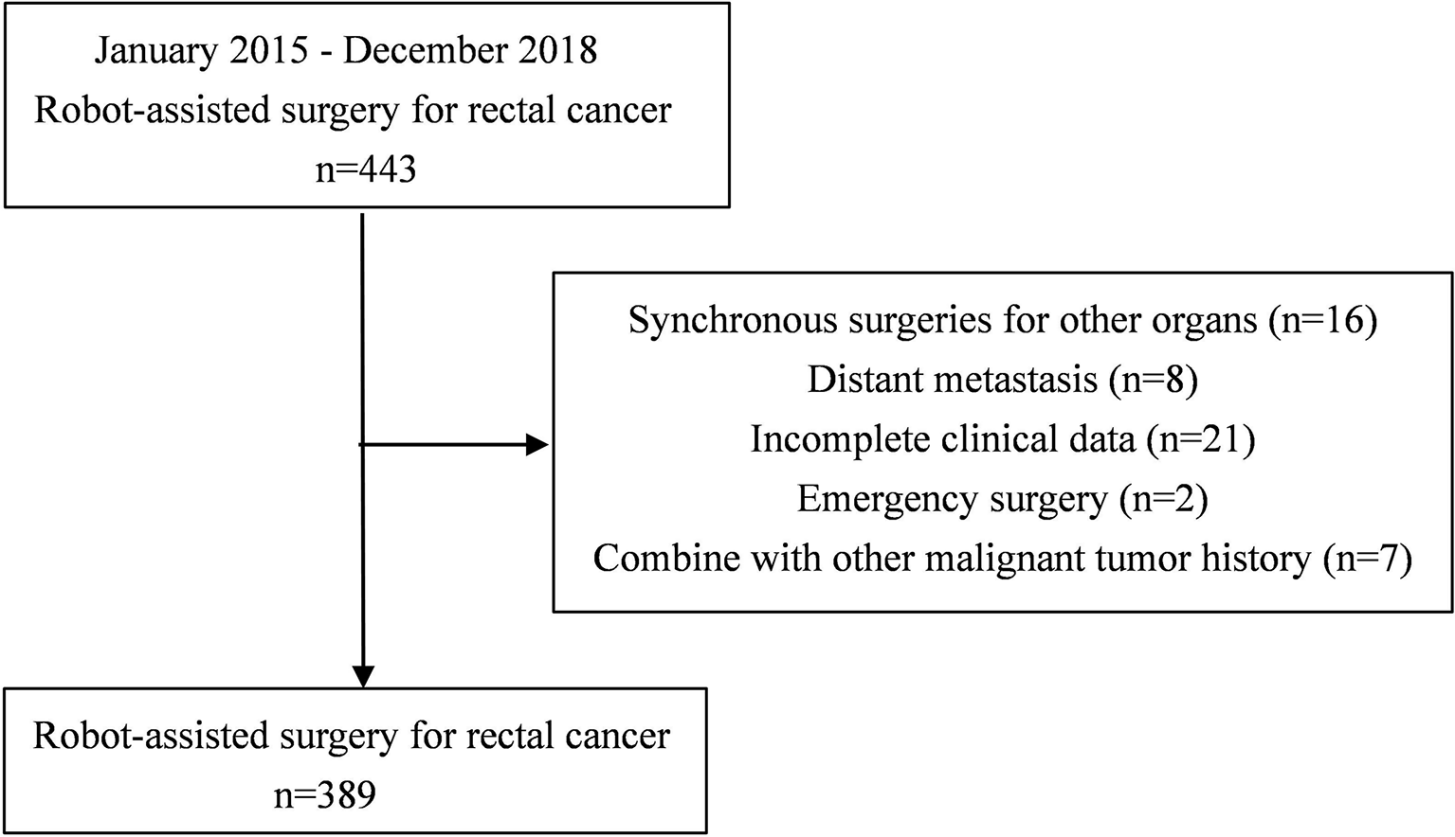
Flowchart of patient selection.

**Figure 3:**
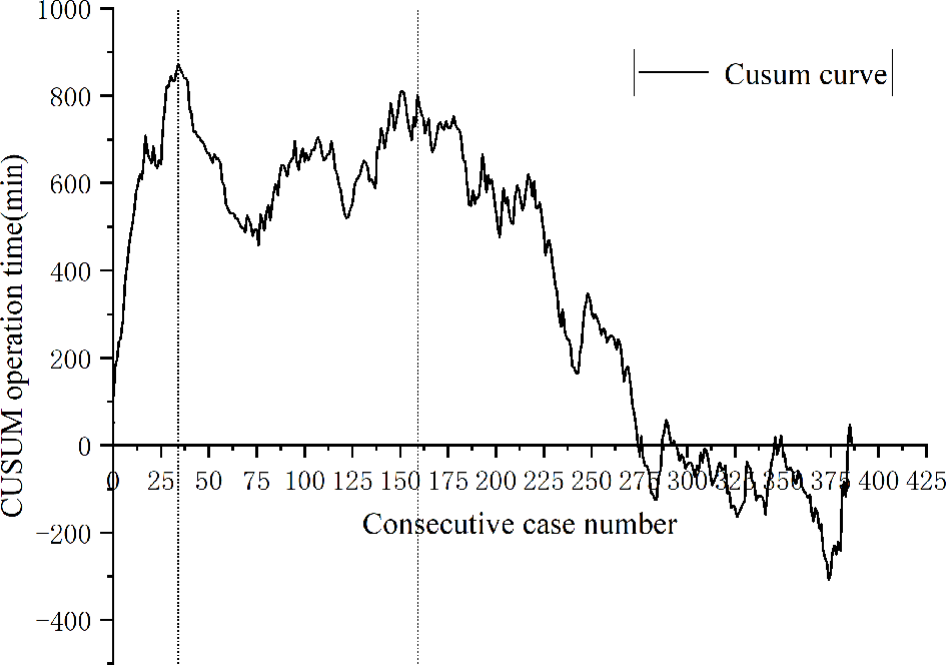
Cumulative sum analysis for operation time for a series of 389 consecutive patients. The X axis indicates consecutive cases, and the Y axis indicates the CUSUM score for operation time. Vertical lines indicate the turning points wherein the learning phase changes. Three learning phases were identified through turning points of the CUSUM curve. The learning phase shows an increasing trend of operation time until 34 cases, with a subsequent decrease. The curve resurged with increased operation time to 151 cases (challenge phase) and then gradually decreased during the expert phase.

**Figure 4:**
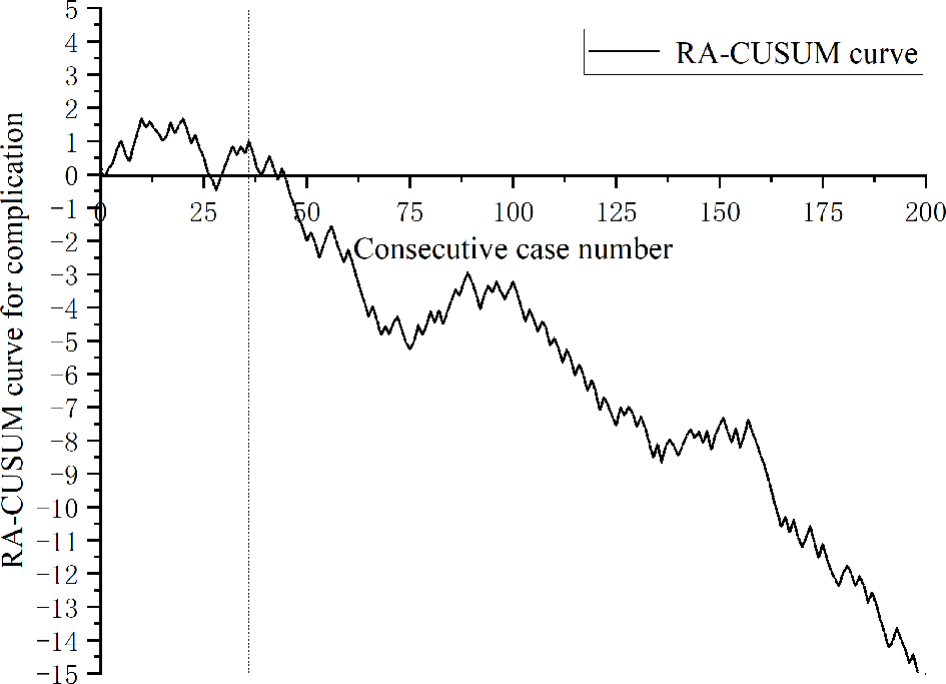
Risk-adjusted cumulative sum analysis for postoperative complications (CD ≥ II) for a series of 389 consecutive patients. The X axis indicates consecutive cases, and the Y axis indicates the RA-CUSUM score for postoperative complications. Vertical lines indicate the turning points wherein the learning phase changes. Two learning phases were identified through turning points of the RA-CUSUM curve. The learning phase shows an increasing trend of morbidity until 36 cases, with a subsequent continuous decrease from 37 to 389 cases during the mastery phase.

A total of 443 patients who underwent robotic-assisted total mesorectal excision for rectal cancer were initially enrolled in this study. Based on the selection criteria, 389 patients were included in the final analysis (**Figure 2**). The baseline characteristics and perioperative outcomes are presented in **Table 1**. The learning curve of operation time is plotted in **Figure 3**. The turning points were observed at 34 cases and 151 cases; the learning curve of postoperative complications (CD≥ grade II) is displayed in **Figure 4**, and only one turning point was observed at 36 cases. Based on this turning point, the learning curve was divided into the learning phase (cases one to 36) and mastery phase (cases 37 to 389).

**Table 1:** Baseline characteristics and perioperative outcomes of included patients.

Variables	N = 389
Age (years)	59.7 ± 13.0
Sex (man/women, n (%))	237 (60.9)/152 (39.1)
ASA score (I/II/III, n (%))	122 (31.4)/182 (46.8)/85 (21.9)
BMI (kg/m^2^)	22.7 ± 3.0
CEA (ug/L)	4.5 (0.4-719.6)
CA 19.9 (U/ml)	8.2 (0.5-783.3)
Tumor location (cm)	6.0 ± 2.6
Neoadjuvant therapy n (%)	68 (17.5)
Operation method (LAR/APR/ISR, n%)	285 (73.3)/69 (17.7)/35 (9.8)
NOSES n (%)	58 (14.9)
Stoma n (%)	79 (20.3)
Conversion n (%)	3 (0.8)
Operation time (min)	160.6 ± 30.1
Blood loss (ml)	180.5 ± 124.1
Postoperative hospital stay (d)	9.1 ± 4.2
Postoperative complications n (%)	81 (20.8)
anastomosis leakage n (%)	35 (10.9)
rectovaginal fistula n (%)	2 (0.5)
intestinal obstruction n (%)	3 (0.8)
wound infection n (%)	15 (3.9)
presacral infection n (%)	5 (1.3)
pulmonary infection n (%)	7 (1.8)
intra-abdominal infections n (%)	3 (0.8)
urinary complications n (%)	9 (2.3)
bleeding n (%)	2 (0.5)
Reoperation n (%)	4 (1.0)
Number of resected lymph nodes	14.1 ± 5.2
Numbers of insufficient harvested lymph nodes	31 (8.0)
Positive DRM n (%)	0 (0)
Positive CRM n (%)	2 (0.5)
TNM stage (I/II/III, %)	44 (11.3)/198 (50.9)/147 (37.8)

ASA, American Society of Anesthesiologists; BMI, body mass index; CEA, carcinoembryonic antigen; NOSES, natural orifice specimen extraction surgery; LAR, low anterior resection; APR, abdominoperineal resection; ISR, intersphincteric resection; DRM, distal resection margin; CRM, circumferential resection margin.

### Patient Characteristics for the Two Phases

Before PSM, the mastery phase had more patients with older age, lower tumor location, and neoadjuvant therapy; after matching, the two phases exhibited similar characteristics (**Table 2**).

**Table 2:** Patient characteristics for two phases before and after propensity score matching.

Characteristics	Before matching			After matching		
	Learning phase (n = 36)	Mastery phase (n = 353)	P-value	Learning phase (n = 36)	Mastery phase (n = 36)	P-value
Age (years)	54.9 ± 14.7	59.9 ± 12.9	0.030	54.9 ± 14.7	55.0 ± 15.2	0.987
Sex n (%)			0.293			0.814
man	19 (52.8)	218 (61.8)		19 (52.8)	18 (50.0)	
women	17 (47.2)	135 (38.2)		17 (47.2)	18 (50.0)	
ASA score ≥III	4	81	0.103	4	4	0.708
BMI (kg/m^2^)	23.0 ± 3.1	22.6 ± 3.0	0.452	23.0 ± 3.1	22.8 ± 3.1	0.804
CEA (ug/L)	3.9 (0.9-43.2)	4.64 (0.4-719.6)	0.632	3.0 (0.9-43.2)	6.4 (0.9-535.30)	0.193
CA19.9 (U/ml)	7.4 (0.6-52.2)	8.3 (0.6-783.3)	0.612	4.4 (1.0-52.3)	8.7 (1.5-603.2)	0.189
Tumor location (cm)	7.1 ± 2.6	5.9 ± 2.6	0.011	7.1 ± 2.6	7.0 ± 2.4	0.780
Neoadjuvant therapy n (%)	2 (5.6)	66 (18.7)	0.048	2 (5.6)	5 (13.9)	0.708

### Perioperative Outcomes for the Two Phases

The surgical methods were similar between two phases. Compared to learning phase, the mastery phase showed shorter operation time (185.2 ± 30.3 vs. 150.0 ± 22.8; P = 0.000), less intraoperative blood loss (196.2 ± 112.6 vs. 140.3 ± 50.2; P = 0.010), shorter postoperative hospital stay (12.3 ± 6.3 vs. 8.9 ± 3.3; P= 0.007), and fewer postoperative complications (38.9% vs. 16.7%, P = 0.033). One patient underwent reoperation in learning phase due to anastomotic leakage and underwent temporary ileostomy. The postoperative pathological results, including the number of resected lymph nodes, numbers of insufficient number of harvested lymph nodes, positive DRM, positive CRM, and TNM stage, were comparable between the two phases (**Table 3**).

**Table 3:** Perioperative outcomes for two phases after propensity score matching.

Variable	Learning phase (n = 36)	Mastery phase (n = 36)	P-value
Operation method			0.474
LAR n (%)	28 (77.8)	26 (72.2)	
APR n (%)	8 (22.2)	7 (19.4)	
ISR n (%)	0 (0)	3 (8.3)	
NOSES n (%)	0 (0)	3 (8.3)	0.238
Stoma n (%)	7 (19.4)	6 (16.7)	0.759
Conversion n (%)	2 (5.56)	0 (0)	0.473
Operation time (min)	185.1 ± 30.3	150.0 ± 22.8	0.000
Blood loss (ml)	196.2 ± 112.6	140.3 ± 50.2	0.008
Postoperative hospital stay (d)	12.3 ± 6.3	8.9 ± 3.3	0.007
Postoperative complications n (%)	14 (38.9)	6 (16.7)	0.033
anastomosis leakage	5 (17.9)	2 (6.9)	
wound infection	3 (8.3)	1 (2.8)	
presacral infection	1 (2.8)	1 (2.8)	
pulmonary infection	2 (5.6)	1 (2.8)	
urinary complications	2 (5.6)	1 (2.8)	
bleeding	1 (2.8)	0 (0)	
Reoperation n (%)	1 (2.8)	0 (0)	1.000
Number of resected lymph nodes	13.0 ± 4.4	13.7 ± 4.8	0.783
Numbers of insufficient harvested lymph nodes	3 (8.3)	1 (2.8)	0.607
Positive DRM n (%)	0 (0)	0 (0)	1.000
Positive CRM n (%)	0 (0)	0 (0)	1.000
TNM stage			0.120
I n (%)	8 (22.2)	2 (5.6)	
II n (%)	19 (52.8)	24 (66.7)	
III n (%)	9 (25.0)	10 (27.8)	

### Survival Outcomes for the Two Phases

**Figure 5:**
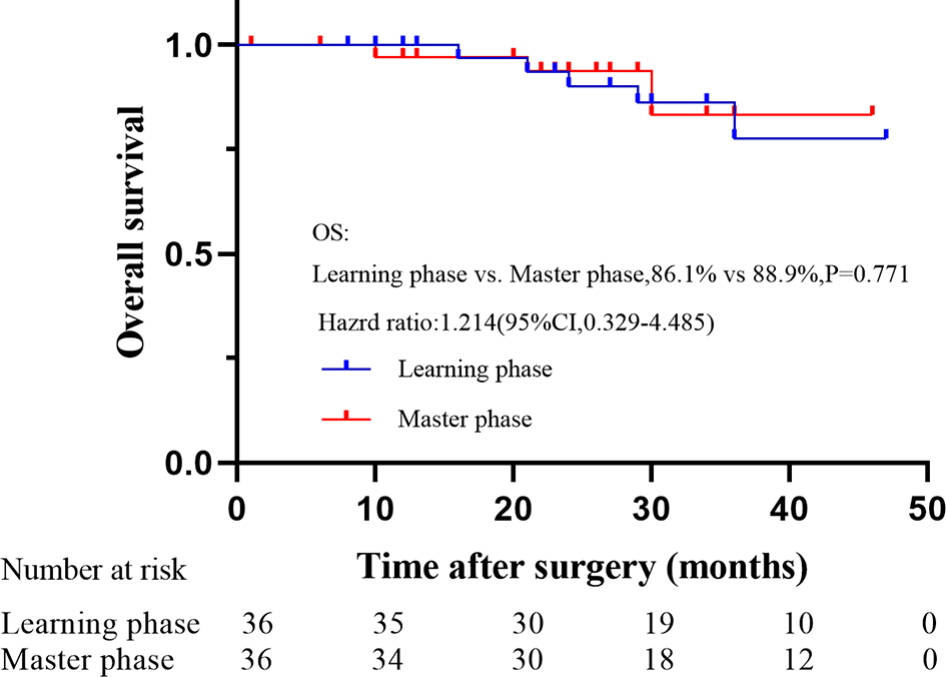
Overall survival rate between two phases after propensity score matching.

**Figure 6:**
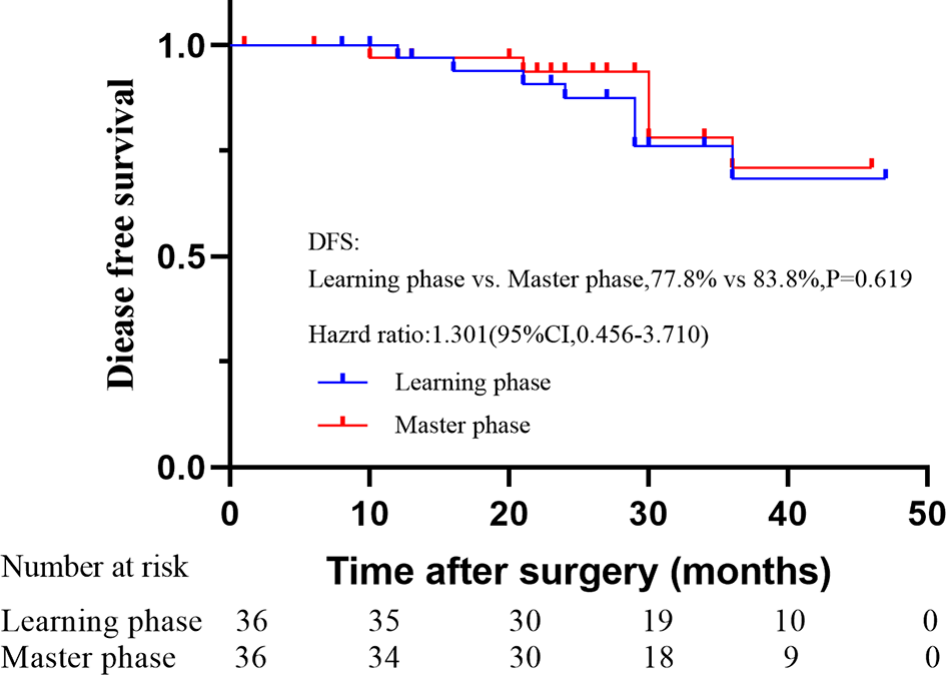
Disease-free survival rate between two phases after propensity score matching.

With a median follow-up of 35 months (range: 23 to 49 months), the OS and DFS were 86.1% and 77.8% in the learning phase and 88.9% and 83.8% in the mastery phase, and no significant difference was observed between the two phases (HR=1.214, 95% CI: 0.329-4.485, P=0.771; HR=1.301, 95% CI: 0.456-3.710, P=0.619; **Figures 5**, **6**).

## Discussion

In the development of any new medical technology there exists a learning curve; in surgical fields, the learning curve represents the growing process of surgical techniques. Realizing the learning process cannot only overcome the fear when initially implementing robotic-assisted surgery, but also help surgeons to get through the learning curve in a safe and effective manner and improve the clinical outcomes during the learning process. In this study, we included 389 consecutive patients undergoing robotic-assisted total mesorectal excision for rectal cancer, which was performed by a single experienced laparoscopic surgeon. The results showed that the number of patients needed to overcome the postoperative complications was 36 and that needed to overcome the learning curves of operation time was 34 in robotic-assisted laparoscopic surgery for rectal cancer. Based on RA-CUSUM, the learning process was divided into the learning phase (1st-36th^t^ cases) and mastery phase (37th-389th cases). The operation time, intraoperative blood loss, postoperative hospital stay, and postoperative complications were reduced in the mastery phase, and the long-term outcomes were not significantly different between the two phases.

Operation time is the most widely used index to evaluate the learning curve, which reflects the adoptability and competitiveness of the surgical technique (16, 25). Parascandola et al. (26) analyzed the operation time learning curve of 502 patients who underwent robotic-assisted laparoscopic rectal cancer surgery. The results showed that plateau performance was achieved in 55-65 cases. Olthof et al. (14) evaluated the learning curve of 100 consecutive robotic-assisted laparoscopic rectal cancer surgeries. The results showed that the operation time greatly decreased over the first 40 cases. However, in our study, two turning points were found on the CUSUM curve. The first turning point is similar to the series by Olthof et al, but rather different from the study of Parascandola et al. In our study, after performing 34 operations, the surgeon became proficient and gradually mastered the robotic skill. The second turning point was observed in 151 cases. During this period, the patients with low rectal tumors who underwent NOSES gradually increased. With the enhancement of surgical maturity, team cooperation, and the self-confidence of the surgeon, the surgeon began to challenge some difficult operations during this period, such as robotic-assisted intersphincteric resection of rectal cancer and NOSES. Therefore, we further divided the mastery phase into the challenge phase (35th-151st cases) and expert phase (152nd-389th cases). After passing the challenge phase, the surgeons were skilled in various types of robotic-assisted surgery for rectal cancer. The surgeon was the first surgeon to carry out robotic-assisted laparoscopic rectal cancer surgery in our province. To date, thousands of robotic-assisted laparoscopic surgeries have been performed. According to our experience, we suggest that patients with a high tumor position and early tumor stage can be selected when robotic-assisted laparoscopic rectal cancer surgery is initially implemented to get through the learning phase in a safe and smooth way. With the increase in proficiency, the surgeon then challenges some patients with low rectal cancer and NOSES.

Although operative time is the most widely used indicator for analyzing learning curves, it only represents the surgical efficiency, which is deficient from the patient point of view (25, 27). The safety of the surgical technique is also important, that is, the new technique should not incur an added risk of postoperative complications (23). Therefore, learning-associated postoperative complications also need to be understood along with procedure proficiency. In this study, the postoperative complications during the learning period of robotic-assisted surgery for rectal cancer were 38.9%, which was less than that during the learning period of laparoscopic surgery (52%) (4). At present, published reports all defined surgical failure based on markers including conversion, R1 resection, CD ≥ 3, insufficient lymph nodes harvested, and local recurrence to evaluate the learning curve of surgical outcomes in robotic rectal surgery. Lee et al. (28) enrolled 506 consecutive patients to analyze the learning curve of surgical failure in robotic-assisted laparoscopic surgery for rectal cancer, and their results showed that surgical outcomes improved after the 177th case. Park et al. (29) analyzed the clinical outcomes of 130 consecutive patients who underwent robotic low anterior resection, and the learning curve of surgical failure showed that feasible perioperative outcomes were achieved at the 75th case. Kim et al. (30) included 167 patients who underwent robotic TME for rectal cancer to analyze the learning curve of surgical failure, and their results showed that the adverse events began to decrease after 32 cases. As far as we are aware, this is the first study that adopted postoperative complications as an independent variable for learning curve analysis of robotic-assisted rectal surgery. The results of our study indicated that the number of patients required to overcome the learning curve of postoperative complications was 36. These published results are quite different from ours, which may be caused by inconsistent evaluation indices and surgical methods. Park et al. (29) and Kim et al. (30) adopted hybrid surgical techniques when initially implementing robotic surgery, whereas Lee et al. (28) used complete robotic surgery. Our results were similar to Lee et al., who adopted complete robotic surgery when initially implementing robotic-assisted surgery. Based on the RA-CUSUM, the learning process was divided into the learning phase (1st-36th cases) and the mastery phase (37th-389th cases), and the operation time, intraoperative blood loss, postoperative hospital stay, and postoperative complications were reduced in the mastery phase. However, in challenge phase divided by CUSUM analysis, we started to challenge some anal-preserving operation for ultralow rectal cancer and NOSES, the operation time increased, but the postoperative complications did not increase, which may be attributed to the increase in surgical proficiency and team cooperation. Coupled with the advantages of the robot platform, increasing experience in the multimodality treatment of rectal cancer, such as MDT discussion, patient selection, and complication management, the implementation of ultralow anus-preserving surgery and NOSES in the later phase did not increase the incidence of postoperative complications.

To our knowledge, this is the first study to evaluate whether the learning period of robotic-assisted laparoscopic surgery for rectal cancer affects the long-term survival of patients. Our results showed that the number of lymph nodes harvested, positive CRM, positive DRM, and long-term oncologic results were not significantly different between the learning phase and mastery phase after PSM. Because the surgeon had rich experience in laparoscopic rectal cancer surgery, the principle of tumor radical resection was followed when robotic surgery was initially adopted, so the learning period seemly did not have an obvious effect on the long-term survival of patients.

Although the sample size included in this study was large and used postoperative complications (CD≥ grade II) as the end point for the first time to evaluate the learning curve of robotic-assisted laparoscopic surgery for rectal cancer, there were also certain limitations in this study. First, this was a retrospective study based on a single experienced laparoscopic surgeon (TY. L), the collected data may be biased, and the generalizability of the results may be reduced. Second, the impacts of surgeons with different laparoscopic surgery experience on the learning curve were not evaluated in this study. Finally, the sample sizes and follow-up time within the learning phase were limited, and the impact of the learning period on the long-term oncologic results still needs to be further evaluated by a multicenter study.

## Conclusions

An experienced laparoscopic surgeon carried out robotic-assisted total mesorectal excision for rectal cancer, the number of patients needed to overcome the learning curve of operation time was 34, the number needed to overcome postoperative complications was 36, and the surgical outcomes improved after 36 cases. The learning curve seemingly did not have an obvious impact on long-term oncologic outcomes.

## Data Availability Statement

The original contributions presented in the study are included in the article/supplementary material. Further inquiries can be directed to the corresponding authors.

## Ethics Statement

The studies involving human participants were reviewed and approved by The Ethics Committee of The First Affiliated Hospital of Nanchang University. The patients/participants provided their written informed consent to participate in this study.

## Author Contributions

TYL and JS designed the study, BT wrote the main manuscript text; GG prepared **Figures 1**–**6**, TL prepared **Tables 1**–**3**. All authors have read and approved the manuscript.

## Conflict of Interest

The authors declare that the research was conducted in the absence of any commercial or financial relationships that could be construed as a potential conflict of interest.

## Publisher’s Note

All claims expressed in this article are solely those of the authors and do not necessarily represent those of their affiliated organizations, or those of the publisher, the editors and the reviewers. Any product that may be evaluated in this article, or claim that may be made by its manufacturer, is not guaranteed or endorsed by the publisher.

## Abbreviations

TME, total mesorectal excision; ASA, American Society of Anesthesiologists; BMI, body mass index; CEA, carcinoembryonic antigen; NOSES, natural orifice specimen extraction surgery; DRM, distal resection margin; CRM, circumferential resection margin; OS, overall survival; DFS, disease free survival; LAR, low anterior resection; APR, abdominoperineal resection; CUSUM, cumulative sum; RA-CUSUM; risk-adjusted cumulative sum.
